# Gut Microbiota and COVID-19: Unraveling the Gut–Lung Axis and Immunomodulatory Therapies

**DOI:** 10.1021/acsinfecdis.5c00250

**Published:** 2025-06-05

**Authors:** Maria Cidinaria Silva Alves, Mireli Santana Rego, Ruana Carolina Cabral da Silva, Rousilândia de Araújo Silva, Igor Eduardo Silva Arruda, Sérgio de Sá Leitão Paiva-Júnior, Valdir de Queiroz Balbino

**Affiliations:** † Laboratório de Bioinformática e Biologia Evolutiva, Centro de Biociências, Departamento de Genética, 28116 Universidade Federal de Pernambuco, Recife, Pernambuco 50670-423, Brazil; ‡ Laboratório de Pesquisa em Ciências da Saúde, 186079 Universidade Federal da Grande Dourados, Dourados, Mato Grosso do Sul 79825-070, Brazil; § Núcleo de Controle de Qualidade de Medicamentos e Correlatos, Departamento de Ciências Farmacêuticas, 28116 Universidade Federal de Pernambuco, Recife, Pernambuco 50670-423, Brazil

**Keywords:** COVID-19, gut flora, immune responses, intestinal dysbiosis, microbiota, therapeutic approaches

## Abstract

The gut flora modulates immune responses and influences COVID-19 severity. SARS-CoV-2 disrupts the gut microbiota, causing dysbiosis, increased intestinal permeability, and systemic inflammation and worsening clinical outcomes. Dysbiosis correlates with elevated inflammatory markers, such as CRP and PCT, contributing to severe complications. Studies show that COVID-19 patients have reduced beneficial bacteria, such as and *Bifidobacterium* spp., alongside increased opportunistic pathogens. This review explores how gut microbiota impacts COVID-19 through predictive microbial signatures and immunomodulatory mechanisms. Therapeutic strategies, including probiotics, prebiotics, and fiber-rich diets, may restore microbial balance, reduce inflammation, and support recovery. Additionally, we examine the effects of antiviral and immunomodulatory therapies on the gut microbiota and their role in post-COVID-19 rehabilitation. Understanding the gut–lung axis in SARS-CoV-2 pathogenesis may reveal microbiota-targeted treatments to improve outcomes and prevent complications. As the host organ with the highest microbial diversity, the gut plays a crucial role in viral infections and warrants further research.

## Graphical abstract

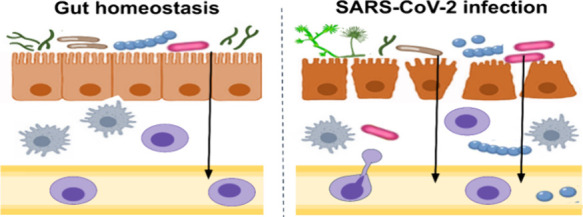

## Introduction

Severe acute respiratory syndrome coronavirus 2 (SARS-CoV-2) infection can be associated with gastrointestinal symptoms,^1^ a feature also observed in other human coronaviruses such as SARS-CoV and MERS-CoV (Middle East respiratory syndrome coronavirus).^2^ Moreover, secondary bacterial infections often occur following viral infections, potentially disrupting the balance of the pulmonary microbiota.^3^ The gut microbiota, composed of trillions of microorganisms, plays critical roles in host metabolic and immune homeostasis. Studies have shown that alterations in the composition of this microbial community are associated with metabolic diseases such as obesity and diabetes.^4^ Additionally, viral infections can dysregulate the gut microbiota, favoring the growth of pathogenic bacteria and reducing the presence of beneficial microorganisms (Figure 1).^5^ In certain contexts, the interaction between viruses and bacteria can exacerbate inflammatory conditions and worsen diseases.^6^ One example is the influence of bacteria-induced epigenetic modifications on the gene expression of viruses with latent infections, such as Kaposi’s sarcoma-associated herpesvirus, Epstein–Barr virus (EBV), and HIV.^7^ These modifications can disrupt viral latency, leading to reactivation of viral production. In HIV-positive individuals, immunosuppression facilitates the growth of opportunistic microorganisms, which contribute to the progression of acquired immunodeficiency syndrome (AIDS).^7^

**Figure 1:**
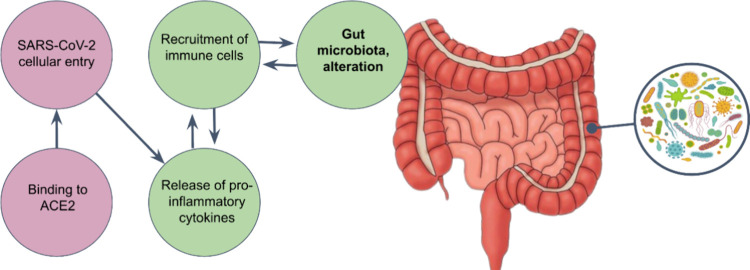
Binding of SARS-CoV-2 Spike proteins to the ACE2 receptor expressed in enterocytes facilitates viral entry, triggering intestinal inflammation through the release of proinflammatory mediators and the recruitment of immune cells, leading to alterations in the gut microbiota.

Respiratory viral infections, including SARS-CoV-2, can trigger exacerbated immune responses, such as cytokine storms, the intensity of which may be modulated by the gut microbiota.^8^ Although research on the relationship between respiratory viruses and gut microbiota is growing, specific studies on the impact of SARS-CoV-2 remain limited. Given the established interaction between other respiratory viruses and the gut microbiota, understanding the mechanisms by which this microbial community influences COVID-19 (coronavirus disease 2019) severity becomes essential.^9^

Accordingly, this review aims to critically examine the current body of evidence regarding the impact of SARS-CoV-2 infection on the gut microbiota, with an emphasis on its modulatory effects on host immune responses and its potential role in the pathophysiology of COVID-19-related complications. By compiling and critically analyzing data from previously published studies, this review intends to contribute to the understanding of the mechanisms associated with severe clinical outcomes and to highlight emerging perspectives for the development of microbiota-targeted therapeutic strategies (Table 1).^10–24^

**Table 1:** General Influence of Gut Microbiota on SARS-CoV-2 Infection

**factor**	**effect on SARS-CoV-2 infection**	**refs**
decrease in *Bifidobacterium* and *Lactobacillus*	weakens intestinal barrier, increases inflammation	^10–12^
gut dysbiosis	increases intestinal permeability, worsens COVID-19 severity	^13–15^
short-chain fatty acid (SCFA) production	modulates immune response, potential protection against severe infection	^16–18^
antibiotic use	reduces microbial diversity, negatively impacts recovery	^19–21^
probiotics and prebiotics	potential beneficial effects on immune regulation and inflammation reduction	^22–24^

### Gut Microbiota and the Gut–Lung Axis

The human gut microbiota comprises approximately 10^14^ microorganisms including bacteria, archaea, viruses, and fungi. In healthy individuals, four main bacterial phyla predominate: Actinobacteria, Firmicutes, Proteobacteria, and Bacteroidetes.^25,26^ While the colon harbors a dense and diverse microbial community, with prominent families such as Bacteroidaceae, Prevotellaceae, Rikenellaceae, Lachnospiraceae, and Ruminococcaceae, the small intestine contains a lower microbial load and is dominated by fast-growing, facultative anaerobes like Streptococcaceae, Lactobacillaceae, and Enterobacteriaceae, which are adapted to higher oxygen levels and rapid nutrient turnover.^27–29^ The gut microbiota plays essential roles in maintaining host homeostasis by contributing to protective, trophic, and metabolic functions.

Although intestinal microorganisms benefit from the host, in terms of habitat and nutrition, they play a crucial role in regulating various physiological functions, including digestion and immune system modulation. Alterations in the composition of this microbiota, known as gut dysbiosis, have been associated with several diseases, such as inflammatory bowel disease,^30^ depression,^31,32^ type 2 diabetes,^33,34^ and cardiovascular diseases.^35,36^ Just as in the gut, there is evidence that the lungs also harbor a distinct microbiota.^37^ While Bacteroidetes and Firmicutes dominate the gut, the lung microbiota is primarily composed of Bacteroidetes, Firmicutes, and Proteobacteria.^38^

The interaction between the gut and lung microbiota occurs through the gut–lung axis, a bidirectional communication system in which endotoxins and microbial metabolites can influence lung function via circulation. Similarly, pulmonary inflammation can affect gut microbiota composition.^39^ This cross talk is mediated by various mechanisms, including immune signaling, microbial-derived metabolites such as short-chain fatty acids (SCFAs), and the migration of immune cells primed in the gut to the lungs.^40^ SCFAs, produced by gut commensals during the fermentation of dietary fibers, can modulate systemic immune responses, reduce airway inflammation, and enhance epithelial barrier function in the lungs.^40^ Conversely, respiratory infections or chronic lung diseases may disrupt intestinal homeostasis through systemic inflammation, altered cytokine profiles, and changes in the mucosal immune system. Recent studies have also highlighted the role of gut dysbiosis in exacerbating pulmonary conditions such as asthma, chronic obstructive pulmonary disease (COPD), and COVID-19, reinforcing the therapeutic potential of targeting the gut microbiota to support respiratory health.

Emerging evidence suggests that individuals with imbalanced gut microbiota, characterized by reduced microbial diversity and loss of beneficial bacteria, are more susceptible to respiratory infections and may experience worse clinical outcomes. For example, alterations in the gut microbiome have been associated with increased susceptibility to influenza and RSV infections and with heightened inflammatory responses in the lungs. In the context of COVID-19, several studies have demonstrated that gut dysbiosis correlates with disease severity, prolonged viral shedding, and systemic inflammation.^39,40^ On the other hand, chronic respiratory conditions can impair gut barrier integrity, increase intestinal permeability (“leaky gut”), and promote the translocation of microbial products into the bloodstream, further fueling systemic inflammation and dysbiosis.^41^ These findings underscore the critical interplay between the gut and lungs in shaping immune responses, disease progression, and therapeutic strategies for both gastrointestinal and respiratory disorders.^42^

This interconnection suggests that SARS-CoV-2 may have effects on the gut microbiota. Indeed, studies indicate that respiratory infections can alter the microbial composition of the gut.^43^ In the context of COVID-19, pneumonia and progression to acute respiratory distress syndrome (ARDS) are severe complications, particularly in older adults and immunocompromised individuals.^44^ Clinical and experimental evidence suggests that the gut microbiota plays a role in the pathogenesis of sepsis and ARDS.^45^ A reduction in gut bacterial diversity can trigger dysbiosis, contributing to the worsening of these conditions.^46^

Older adults typically present a less diverse gut microbiota, with a notable reduction in beneficial microorganisms such as *Bifidobacteria*.^47^ This dysbiosis, common in aging and often worsened by comorbidities, has been associated with poorer COVID-19 outcomes. Increased systemic inflammation in this group may compromise intestinal barrier integrity, allowing bacterial metabolites and toxins to enter the circulation and intensify disease severity.^48,49^ These findings suggest that the gut–lung axis plays a relevant role in the clinical progression of COVID-19 among the elderly, reinforcing the link between microbial imbalance and heightened vulnerability to severe manifestations.

### The Influence of Gut Microbiota on Immunity

The interactions between the host and microbiota are complex, numerous, and interdependent. Evidence indicates that the gut microbiota plays an essential role in regulating the function and development of the immune systems.^50^ Commensal microorganisms in the gut secrete antimicrobial peptides, compete for nutrients and ecological niches, and promote homeostasis maintenance.^51^

The relationship between gut microbiota and immune homeostasis is dynamic and constitutes an area of intense research in infectious diseases. Signals derived from these microorganisms modulate the pro- and anti-inflammatory responses of immune cells, affecting susceptibility to various diseases.^52^ The balance between regulatory T cells and proinflammatory Th17 cells is fundamental for intestinal immunoregulation and is largely influenced by commensal microbiota.^53^

In the context of viral infections, such as those caused by SARS-CoV-2, a balanced gut microbiome can be crucial for an efficient immune response, preventing excessive reactions that could compromise vital organs such as the lungs. Proper regulation of the immune response is critical since both hyperactivity and immune insufficiency can exacerbate clinical complications, including pneumonia and ARDS, which are common in severe viral infections.

The gut microbiota acts as a source of microorganism-associated molecular patterns (MAMPs) and pathogen-associated molecular patterns (PAMPs), which are recognized by pattern recognition receptors (PRRs) such as nucleotide-binding oligomerization domain (NOD) receptors and toll-like receptors (TLRs).^54^ These receptors detect MAMPs and PAMPs, triggering specific immune responses depending on the type of receptor, ligand, cell involved in the response.

PRR training in innate immune cells that express microbial or nonmicrobial ligands from the gut is an essential mechanism of protection independent of adaptive immunity in secondary infections and pathogenic exposures. The gut microbiota also secretes metabolites and immunomodulatory molecules, such as short-chain fatty acids (SCFAs), including butyrate, acetate, and propionate, as well as secondary bile acids, produced by microorganisms like *Bacteroides*, *Lactobacillus*, and *Bifidobacterium*.^55^ These substances interact with receptors in immune cells, such as dendritic cells (DCs) and macrophages, modulating their metabolism and functions.^55–57^

Studies have shown that administering probiotics, such as , to healthy elderly individuals can significantly increase the proportion of mononuclear leukocytes and the cytotoxic activity of NK cells, strengthening immunity.^58^ A balanced gut microbiota composition also directly influences the effectiveness of pulmonary immune responses.^37^ In experimental models, germ-free (GF) mice, which lack gut microbiota, exhibit lower efficiency in eliminating pulmonary pathogens.^59^

Gut dysbiosis, often induced by the indiscriminate use of antibiotics, can compromise immune responses and has been associated with an increased risk of lung cancer in population studies.^60^ Additionally, respiratory infections, such as influenza -virus-induced flu, alter the composition of the gut microbiota, leading to an increase in Enterobacteriaceae and a reduction in *Lactobacillus* and *Lactococcus*.^61^

Given the central role of gut microbiota in immune regulation, the relationship between SARS-CoV-2 and commensal microorganisms in the gut and lungs should be extensively investigated. Understanding these mechanisms may contribute to the development of therapeutic strategies focused on microbiota modulation as a complementary approach to managing the effects of COVID-19.

### Gut Microbiota Alterations Related to COVID-19

The gut microbiota is a complex ecosystem composed of thousands of species whose diversity is shaped by genetic and environmental factors. The interaction between this microbiota and human health has been extensively studied, with evidence indicating its influence on inflammatory conditions, allergies, and respiratory diseases.^62^ In patients affected by COVID-19, in addition to the observed gastrointestinal symptoms, changes in microbiome composition have been reported with potential implications for disease diagnosis and treatment.

The gut microbiome is often called a "virtual" or "forgotten organ" because of its vital role in host physiology and overall health, having evolved over centuries in a symbiotic relationship with the human body.^63,64^ Its composition and diversity are influenced by factors such as diet, culture, and geographical location, which have also been associated with the severity of COVID-19.^65^ The microbiome plays an essential role in regulating the immune system and maintaining homeostasis. However, alterations in this balance may occur due to aging, respiratory viral infections, and chronic diseases, resulting in an increased abundance of Bacteroidetes and a reduction in the presence of Firmicutes.^66^ Studies in murine models have demonstrated that the expression of the angiotensin-converting enzyme 2 (ACE2) receptor in the colon is downregulated by certain Bacteroidetes species, while its interaction with Firmicutes remains unclear. Additionally, Mao et al.^67^ pointed out that comorbidities such as diabetes, cardiovascular diseases, and obesity are also associated with microbiota alterations, potentially contributing to adverse outcomes in COVID-19 patients.^68^

Gut dysbiosis has been linked to various inflammatory and infectious diseases.^69^ Studies indicate the persistence of SARS-CoV-2 genetic material in the feces of patients even after the resolution of respiratory symptoms, suggesting that the gastrointestinal tract may serve as a site of viral replication and raising concerns about possible fecal–oral transmission.^70^ Additionally, an increase in opportunistic bacteria such as , , , and has been identified in patients with positive fecal test results for the virus. In a study involving 30 COVID-19 patients, a reduction in bacterial diversity and an increase in the presence of opportunistic pathogens, including *Streptococcus, Rothia, Veillonella, Erysipelatoclostridium,* and *Actinomyces,* were observed.

Studies show that gut microbiota composition may also be related to COVID-19 severity.^9^ Severe patients exhibited a higher abundance of *Coprobacillus*, , and , while beneficial species such as Bacteroidetes and showed a negative correlation with disease severity. Four *Bacteroides* species (, , , and ) demonstrated a negative association with fecal SARS-CoV-2 viral load.^70^

Another relevant factor is the relationship between gut dysbiosis and inflammatory cytokine levels. Some bacterial species, such as and , positively correlated with IL-1β, IL-6, and CXCL8, while , , and showed a negative correlation with TNF-α and CXCL10.^71^ Additionally, butyrate producers such as and were significantly reduced in COVID-19 patients, while opportunistic pathogens such as *Enterococcus* and Enterobacteriaceae were increased.^72^ Altered microbiota was also identified in children with Kawasaki disease, suggesting a possible link between SARS-CoV-2 infection and dysbiosis.^73^

The relationship between the gut microbiota and inflammatory markers was also evidenced in proteomic studies. In a study by Gou et al.,^74^ blood biomarkers were associated with the risk of exacerbated inflammation in elderly COVID-19 patients. Furthermore, the presence of *Lactobacillus* species correlated with IL-10 and more favorable outcomes, while *Klebsiella, Streptococcus,* and were associated with disease severity. Metabolomic studies suggest that gut dysbiosis may compromise intestinal barrier integrity, favoring microbial translocation and increasing systemic inflammation (Figure 2).^74,75^

**Figure 2:**
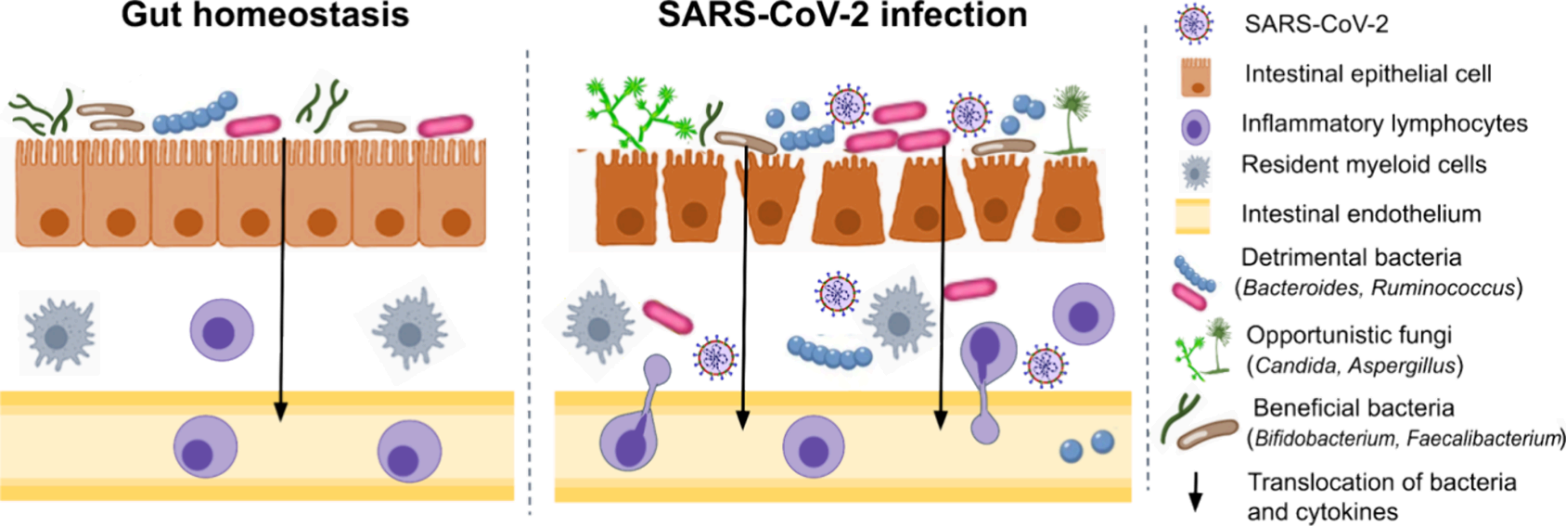
SARS-CoV-2 infection induces microbial dysbiosis and intestinal inflammation. Infection of intestinal epithelial cells by SARS-CoV-2 triggers a proinflammatory immune response, leading to the infiltration of inflammatory lymphocytes and disruption of the intestinal barrier. This imbalance in homeostasis allows the overgrowth of pathogenic bacteria, resulting in dysbiosis, while the compromised intestinal barrier facilitates bacterial translocation, exacerbating inflammation. Additionally, opportunistic fungal infections have been observed in some patients, which further contribute to intestinal dysfunction.

Research conducted by Moreira-Rosário et al.^76^ reinforces the hypothesis that alterations in gut microbiota are linked to COVID-19 progression. The researchers identified a reduction in the *Firmicutes-to-Bacteroidetes* ratio as well as in the presence of butyrate-producing bacteria from the Lachnospiraceae family, such as *Roseburia* and *Lachnospira*. Additionally, a decrease in Actinobacteria (including *Bifidobacteria* and *Collinsella*) and an increase in Proteobacteria were observed in more severe cases of the disease. These findings were corroborated by Yeoh et al.,^71^ who found that the microbiome composition of COVID-19 patients differed significantly from that of healthy individuals, marked by an increase in Bacteroidetes and a reduction in Actinobacteria.

Beyond its relationship with the body’s inflammatory state, studies suggest that and , known for their immunomodulatory roles, exhibit a negative correlation with COVID-19 severity. Conversely, and were positively associated with elevated IL-1β and IL-6 levels. These findings align with observations by Zuo et al.,^70^ who reported a reduction in beneficial gut symbionts such as *Roseburia* and Lachnospiraceae and an increase in opportunistic pathogens, including and , which correlated with disease severity.

The gut microbiota plays a crucial role in infection resistance and the pathogenesis of SARS-CoV-2.^77,78^ Bacteroidetes can modulate the TLR4 pathway, reducing the cytokine storm.^79,80^ Additionally, the microbiota regulates heparan sulfate, inhibiting viral adhesion to the target cells. The microbial metabolite butyrate reduces ACE2 expression, suppresses spike protein activation, and inhibits cell death by downregulating high mobility group box 1 (HMGB1).^81,82^ It also stimulates antiviral responses via TLR7. Ursodeoxycholate blocks viral binding to ACE2 and reduces proinflammatory cytokines. Bacteroidetes species, such as and , restrict viral entry through ACE2 (Figure 3).^83,84^

**Figure 3:**
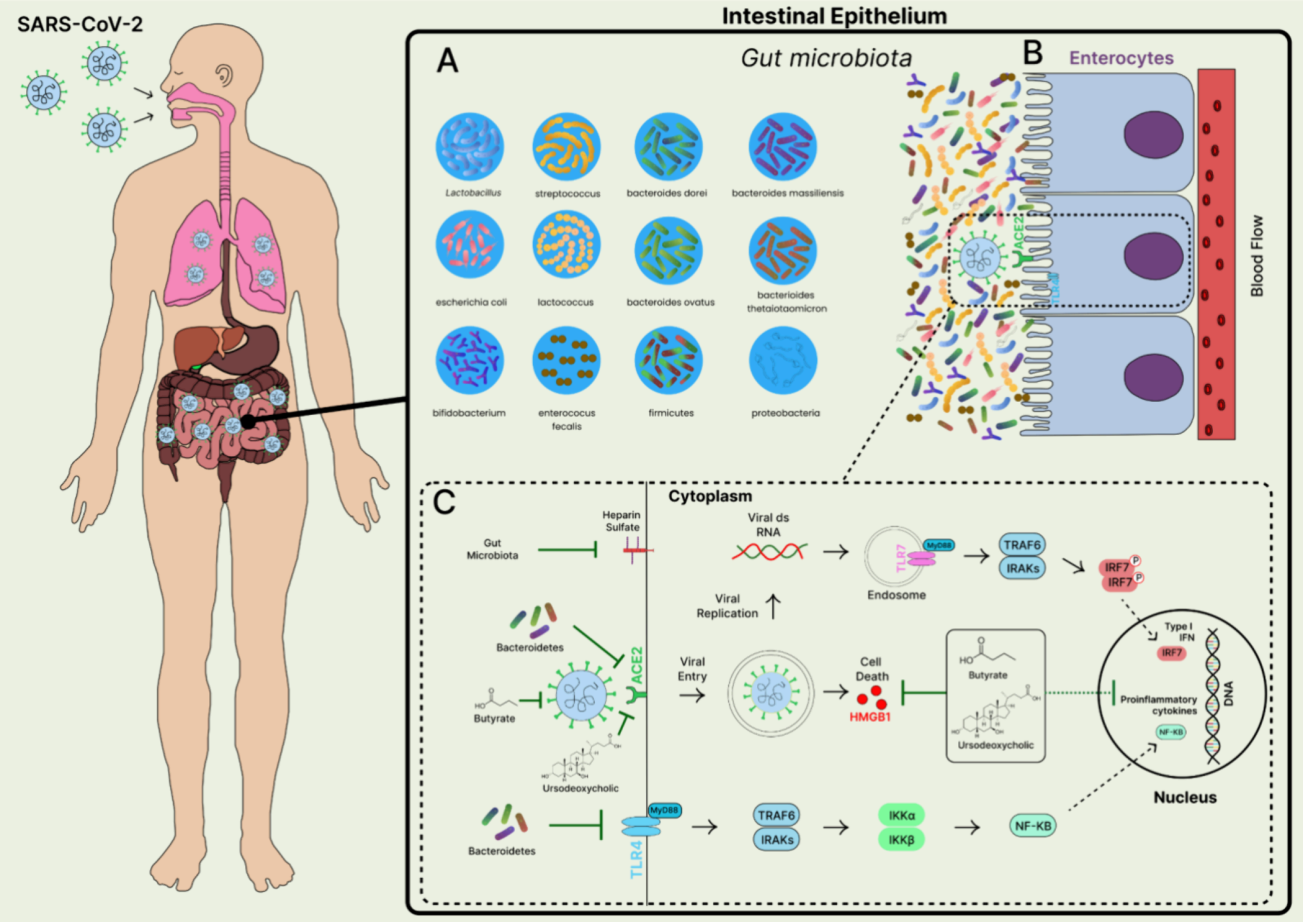
Role of gut microbiota in combating SARS-CoV-2 infection and pathogenesis. (A) Normal gut microbiota. (B)- Enterocytes in the intestinal epithelium express ACE2 receptors, which can bind to SARS-CoV-2. (C) The metabolite butyrate (derived from the gut microbiota) reduces membrane ACE2 expression, suppressing viral spike protein activation and inhibiting virus-induced cell death by downregulating HMGB1. This process triggers antiviral immune responses by activating the TLR7 signaling cascade. Bacteroidetes prevent SARS-CoV-2-induced cytokine proliferation by directing signaling through the TLR4 pathway, thereby blocking ACE2-mediated viral entry. Ursodeoxycholate, another metabolite, exerts anti-SARS-CoV-2 effects by preventing infection through viral binding blockade at ACE2 as well as by restricting proinflammatory cytokine expression. These combined actions alleviate SARS-CoV-2-induced pathology.

Given the relevance of the microbiome in immune modulation and the infection response, its preservation is crucial. COVID-19 patients frequently experience a decline in beneficial bacterial populations and an increase in opportunistic species, a condition that may be exacerbated by pre-existing conditions. This observation opens perspectives for therapeutic interventions, including the use of probiotics and fecal microbiota transplantation, strategies already explored for other infectious diseases. However, the true extent of the microbiota influence on COVID-19 remains incompletely understood, reinforcing the need for further research on its composition, alterations, and potential therapeutic approaches.

### The Importance of the Gut Microbiota in the Severity and Outcomes of COVID-19

The influence of the gut microbiota on the progression and outcomes of COVID-19 has been widely studied, suggesting its potential as a therapeutic target. Clinical evidence indicates that microbiota dysregulation following SARS-CoV-2 infection may impair the immune response, affecting disease severity.^71^

The inflammatory response triggered by the virus impacts the gut microbiota, leading to dysbiosis and alteration of the epithelial barrier integrity. This imbalance increases intestinal permeability, facilitating the entry of toxins and bacterial products into the bloodstream and exacerbating systemic inflammation.^85–87^ Studies analyzing biomarkers in COVID-19 patients have shown elevated concentrations of fatty-acid-binding protein 2, peptidoglycan, and lipopolysaccharide, reinforcing the hypothesis of a compromised intestinal barrier.^88^

COVID-19 patients have exhibited significant changes in microbiota composition, including an increase in *Actinobacteria* spp. and a reduction in *Bacteroides* spp., reflecting an increased Firmicutes-to-Bacteroidetes ratio. The decrease in beneficial bacteria such as *Bifidobacterium* and the rise of opportunistic pathogens like *Brevibacterium* and *Pantoea* have been observed in these patients.^88^

Yeoh et al.^71^ analyzed stool and blood samples from 100 COVID-19 patients, identifying a distinct microbial profile characterized by a reduction in gut commensals such as , , and *Bifidobacterium*. These alterations persisted for up to 30 days after infection in 87 hospitalized patients. Moreover, disease severity correlated negatively with the presence of these microbial species, indicating a lasting effect on the gut microbiota.

Schult et al.^89^ investigated gut microbial profiles across a study population comprising four groups: 108 patients with laboratory-confirmed SARS-CoV-2 infection, 22 individuals who had recovered from COVID-19 and tested negative at the time of sampling, 20 symptomatic pneumonia controls, and 26 age- and gender-matched asymptomatic controls, totaling 251 stool samples. The analysis revealed marked differences between low- and high-risk patients, with predominating in individuals at lower risk of complications, while *Parabacteroides* spp. was more abundant in severe cases. Based on these findings, the researchers identified a set of 12 bacterial species as potential prognostic biomarkers, achieving 94% accuracy in predicting the severity of COVID-19 severity.

Microbial composition was also associated with inflammatory markers, such as white blood cell count, C-reactive protein (CRP), and procalcitonin. In severe and fatal cases, a significant reduction in beneficial species such as , , , and was observed.^89^

Factors such as aging, diet, and comorbidities, including obesity, diabetes, and cardiovascular diseases, play a crucial role in microbiota composition, exacerbating dysbiosis and potentially influencing COVID-19 severity.^90^ In addition to the gut microbiota, alterations have also been observed in the oral microbiota of infected patients, including increased levels of Firmicutes, Actinobacteria, and Bacteroidetes, as well as a higher abundance of lipopolysaccharide-producing bacteria. These changes may serve as an additional source of endotoxins, contributing to the amplification of systemic inflammation observed in more severe cases of the disease.^40^

The prolonged effects of SARS-CoV-2 on the microbiota were evidenced by the persistence of intestinal dysbiosis even after recovery from infection. Species such as Coprobacillus, , and were associated with COVID-19 severity, whereas *Bacteroides* spp. showed negative regulation of ACE2 expression, suggesting a potential protective role against viral infection.^70^

In this context, emerging evidence also points to a significant role of the microbiome in the establishment of viral reservoirs and in the pathogenesis of long COVID, or post-acute sequelae of SARS-CoV-2 infection (PASC). Persistent viral presence in tissues may trigger ongoing immune activation and inflammation, contributing to prolonged symptoms.^91,92^ Alterations in gut microbiota diversity, already linked to acute COVID-19 severity, may persist and increase susceptibility to PASC.^39^ Disruptions in the gut–brain axis have been associated with neuropsychiatric symptoms and chronic inflammation seen in long COVID cases.^93^ Nutritional strategies, such as fiber- and antioxidant-rich diets like the Mediterranean Diet, may help restore microbial balance and reduce systemic inflammation, offering potential to alleviate PASC symptoms.^39^ Although causality remains to be fully elucidated, these findings highlight the need for further investigation into the microbiome’s long-term impact on COVID-19 outcomes.

Microbial metabolites also play a crucial role in modulating the immune system. Species such as , , and produce short-chain fatty acids that can influence the host immune response.^94–96^ Metabolomic profiling of fecal samples from COVID-19 patients revealed significant modifications in metabolites such as monosaccharides, nucleotides, and amino acids, correlating with microbiota alterations.^94^

Therapeutic strategies targeting microbiota modulation have been explored to mitigate the effects of the COVID-19. Fiber-rich diets and probiotic supplements have shown potential in improving the clinical course of the disease.^97^ Ongoing clinical studies are investigating the impact of different probiotic strains on reducing severity and improving post-COVID-19 recovery.

In summary, the gut microbiota plays a central role in modulating the immune response and the progression of COVID-19. Understanding whether microbiota changes are causal or consequential is essential for the development of targeted therapies, including microbiota-modulating interventions, such as probiotics, prebiotics, or fecal microbiota transplantation (FMT). Further studies are needed to delineate the directionality and mechanisms of these interactions, particularly in the context of persistent symptoms and immune dysfunction observed under post-COVID conditions.

## Conclusions

The growing understanding of the interaction between the gut microbiota and immune response in COVID-19 patients highlights the importance of the gut–lung axis in modulating disease severity. Scientific evidence demonstrates that intestinal dysbiosis, characterized by a reduction in beneficial bacteria and an increase in potentially pathogenic microorganisms, is associated with an exacerbated inflammatory response and worse clinical outcomes. Additionally, alterations in the metabolomic profile and intestinal barrier integrity reinforce the impact of the microbiota on the progression of COVID-19 and its associated systemic complications.

In this context, therapeutic strategies aimed at modulating the gut microbiota, such as the use of probiotics, prebiotics, and fiber-rich diets, emerge as promising approaches to reducing systemic inflammation and improving the COVID-19 clinical outcomes. Ongoing clinical trials seek to validate the effectiveness of these interventions in regulating immune responses and mitigating the long-term effects of the disease.

Future research should prioritize the investigation of advanced and integrative strategies for gut microbiota modulation and diagnostic refinement. This includes the development of personalized microbiome-based interventions guided by high-resolution metagenomic, metabolomic, and transcriptomic profiling. Fecal microbiota transplantation (FMT) and the engineering of synthetic microbial consortia specifically designed to correct dysbiosis profiles identified in COVID-19 patients represent promising avenues. Additionally, the application of systems biology frameworks and machine learning algorithms to multiomics data sets may enable the identification of predictive microbial signatures linked to disease severity, therapeutic responsiveness, and long-term complications. These approaches hold potential to advance precision medicine not only in the context of COVID-19 but also in a broader spectrum of microbiota-associated infectious and immune-mediated disorders.

Therefore, continued research into the relationship between microbiota and COVID-19 may significantly contribute to the development of new therapeutic strategies, potentially aiding in the prevention and treatment of viral infections and their complications. Advancing this field could not only enhance the clinical management of COVID-19 but also offer new perspectives for addressing other inflammatory and infectious diseases mediated by the microbiota.
